# The conservative cysteines in transmembrane domain of *At*VKOR/LTO1 are critical for photosynthetic growth and photosystem II activity in *Arabidopsis*

**DOI:** 10.3389/fpls.2015.00238

**Published:** 2015-04-17

**Authors:** Jia-Jia Du, Chun-Yan Zhan, Ying Lu, Hao-Ran Cui, Xiao-Yun Wang

**Affiliations:** State Key Laboratory of Crop Biology, College of Life Science, Shandong Agricultural UniversityTai´an, China

**Keywords:** *At*VKOR, cysteine, disulfide bond, photosystem II, D1 protein

## Abstract

Thylakoid protein vitamin K epoxide reductase (*At*VKOR/LTO1) is involved in oxidoreduction. The deficiency of this compound causes pleiotropic defects in *Arabidopsis thaliana*, such as severely stunted growth, smaller sized leaves, and delay of flowering. Transgenic complementation of wild-type *At*VKOR (VKOR_WT_) to *vkor* mutant lines ultimately demonstrates that the phenotype changes are due to this gene. However, whether *At*VKOR functions in *Arabidopsis* through its protein oxidoreduction is unknown. To further study the redox-active sites of *At*VKOR *in vivo*, a series of plasmids containing cysteine-mutant VKORs were constructed and transformed into *vkor* deficient lines. Compared with transgenic *At*VKOR_WT_ plants, the size of the transgenic plants with a single conservative cysteine mutation (VKOR_C109A_, VKOR_C116A_, VKOR_C195A_, and VKOR_C198A_) were smaller, and two double-cysteine mutations (VKOR_C109AC116A_ and VKOR_C195AC198A_) showed significantly stunted growth, similar with the *vkor* mutant line. However, mutations of two non-conservative cysteines (VKOR_C46A_ and VKOR_C230A_) displayed little obvious changes in the phenotypes of *Arabidopsis*. Consistently, the maximum and actual efficiency of photosystem II (PSII) in double-cysteine mutation plants decreased significantly to the level similar to that of the *vkor* mutant line both under normal growth light and high light. A significantly decreased amount of D1 protein and increased accumulation of reactive oxygen species were observed in two double-cysteine mutations under high light. All of the results above indicated that the conservative cysteines in transmembrane domains were the functional sites of *At*VKOR in* Arabidopsis* and that the oxidoreductase activities of *At*VKOR were directly related to the autotrophic photosynthetic growth and PSII activity of* Arabidopsis thaliana*.

## Introduction

In chloroplasts, disulfide bond formation, a covalent bond between two cysteines, is crucial for the maturation and function of proteins (Jocelyn, 1967; Buchanan and Luan, 2005; Onda, 2013). The majority of protein disulfides in chloroplasts are considered to be one important inert covalent linkage for structure stabilizing modifications (Nagahara, 2011; Romero et al., 2014). However, some regulatory disulfide bonds serve as signaling elements by interchanging their reduced or oxidized states, which plays important roles in photosynthesis, gene expression, signal transduction, and stress resistance (Buchanan and Luan, 2005; Wouters et al., 2010; Cremers and Jakob, 2013; Karamoko et al., 2013; Kieselbach, 2013). Intensive studies regarding the enzymatic reduction of disulfide bonds in redox-regulated proteins in chloroplasts have been conducted, while the reversible process, the formation of a disulfide bond, is yet to be elucidated (Aro and Ohad, 2003; Buchanan and Luan, 2005; Hall et al., 2010; Cremers and Jakob, 2013; Karamoko et al., 2013; Kieselbach, 2013).

A novel thylakoid protein, VKOR, from *Cyanobacteria* and *Arabidopsis thaliana* has been identified to promote disulfide bond formation (Furt et al., 2010; Li et al., 2010; Feng et al., 2011; Karamoko et al., 2011). Compared with *Cyanobacteria* VKOR, plant VKOR has an additional transit peptide at the N-terminus that targets the protein to chloroplasts (Furt et al., 2010; Feng et al., 2011; Wan et al., 2014). Different from VKOR in mammals, both thylakoid VKORs are fusion proteins comprising two domains, a transmembrane/VKOR domain and a soluble thioredoxin-like/Trx-like domain (Oldenburg et al., 2006; Li et al., 2010; Feng et al., 2011). In *Arabidopsis*, the transmembrane domain/VKOR domain of *At*VKOR, which is homologous to VKORs from mammalian, *Synechococcus* sp. and *Mycobacterium tuberculosis*, contains two non-conservative cysteines (Cys46, Cys230) and four conservative cysteines forming two pairs (Feng et al., 2011). One pair is in a separated form (Cys109, Cys116), and the other pair is in a canonical Cys-X-X-Cys motif (Cys195, Cys198). Functional analyses reveal that these two pairs of conservative cysteines are indispensable for the oxidoreductase activities of *At*VKOR in the process of catalyzing disulfide bond formation in *Escherichia coli* (Feng et al., 2011; Karamoko et al., 2011). In* M. tuberculosis*, two pairs of conservative cysteines of *Mtb*VKOR are also found to play critical roles in the formation of disulfide bonds (Wang et al., 2011).

*Arabidopsis* VKOR is also called LTO1, and mutant lines of *vkor* (also called *lto1*) display severely deficient photosynthetic growth and low activities of PSII (Karamoko et al., 2011; Lu et al., 2013). Interestingly, the underlying mechanism of defects in the *vkor* mutant line has not been determined, although transgenic VKOR_WT_ to *vkor* mutant lines demonstrates that the phenotype changes are due to this gene (Karamoko et al., 2011; Lu et al., 2013). In* vitro*, the recombinant Trx-like domain of *At*VKOR can promote the disulfide bond formation of targets in chloroplasts, such as proteins PsbO and FKBP13 (Karamoko et al., 2011; Lu et al., 2013). PsbO, a luminal subunit of PSII, carries a single intramolecular disulfide bond and is essential for the stability of the oxygen-evolving complex (Roose et al., 2010; Roberts et al., 2012). FKBP13, a peptidyl-prolyl *cis*–*trans* isomerase in the thylakoid lumen also contains an essential disulfide bond and its sulfhydryl oxidation plays a vital role in the photosynthetic electron transport chain (Gopalan et al., 2004; Kang et al., 2008). Experiments reveal that another luminal protein regulated by *At*VKOR may be VDE, a luminal enzyme involved in thermal dissipation through the xanthophyll cycle, the activity of which is also dependent on its sulfhydryl oxidation (Bugos and Yamamoto, 1996; Latowski et al., 2004; Yu et al., 2014). Since the redox regulation mechanism of chloroplasts in high plants is complex and vague, we wondered whether *At*VKOR regulated their growth and development through its oxidoreductase activity.

In this investigation, plasmids containing cysteine-mutant VKORs were constructed and transformed into *vkor* mutant lines. Based on the stunted growth phenotype and decreased PSII activity of *vkor* homozygosities containing mutations of single/double conservative cysteines in the VKOR domain, the important role of the oxidoreductase activities of *At*VKOR in photosynthetic growth and PSII activity was confirmed.

## Materials and Methods

### Plant Materials and Growth Conditions

Wild-type *Arabidopsis* ecotype Columbia and the T-DNA insertion *vkor* mutant line have been described in a previous work (Lu et al., 2013). For growth on Murashige and Skoog (MS) medium, the seeds of wild-type and transgenic plants were surface-sterilized with 70% ethanol and 2.6% bleach for 5 and 10 min, respectively. Then, seeds were washed more than five times with sterilized water containing detergent Tween-20. The washed seeds of transgenic or wild-type plants (WTs) were allowed to germinate on MS medium with or without 50 μg ml^-1^ kanamycin. The seeds on plates were stratified for 48 h at 4°C in the dark for synchronized germination. After 2 weeks, the plantlets were transplanted into vermiculite under 120 μmol m^-2^ s^-1^ with short-day conditions (8-h-light/16-h-dark) or long-day conditions (16-h-light/8-h-dark) at a constant temperature of 22°C.

### Plasmids for Plant Transformation

The verified sequences of single/double cysteine-mutant *At*VKORs were fused to plant transformation plasmid pBI121, following the previous operations of the plasmid pBI121 of wild-type *AtVKOR* with full-length cDNA (Lu et al., 2013). The heterozygotic *Arabidopsis* of wild-type Columbia and the *vkor* mutant line were determined by T-DNA specific primers (Forward primer of *AtVKOR*, 5^′^-CTTACCTGCAATGCAATGTTG-3^′^; reverse primer of *AtVKOR*, 5^′^-ACCAGTTTCCAATTCGTGATG-3^′^; T-DNA specific primer, 5^′^-GCGTGGACCGCTTGCTGCAACT-3^′^) and were used for floral dip transformation. Transgenic plants were selected for kanamycin resistance and verified by genomic PCR with specific primers (forward primer for genomic PCR, 5^′^-GGCCATGGAGTCAAAGATTC-3^′^; reverse primer for genomic PCR, 5^′^-CATTGCAGTCGTGATCCC-3^′^). In the next generation, the homozygotes of cysteine-mutant VKORs to* vkor* mutant background were screened by T-DNA specific primers as described above.

### RNA Extraction and Semiquantitative RT-PCR

The leaves of WT, the *vkor* mutant line, and transgenic cysteine-mutant VKORs and *At*VKOR_WT_ plants were used for total RNA isolation using the method described by Lu et al. (2014). The cDNA synthesis was performed according to standard procedures of RevertAid Fist Strand cDNA Synthesis Kit (Fermentas, Canada). In a semiquantitative RT-PCR assay, elongation factor 1-alpha (*EF1-α*) was used as a control for normalization. The PCR cycles were as follows: one cycle of 5 min at 95°C, followed by 28 cycles each of 30 s at 95°C, 30 s at 57°C, and 30 s at 72°C, final cycle of 8 min at 72°C. Forward primer of *EF1-*α, 5^′^-GAGGCTGGTATCTCTAAGGA-3^′^, reverse primer of *EF1-α*, 5^′^-GGAAGTGCCTCAAGAAGAGA-3^′^; forward primer of *AtVKOR*, 5^′^-GTCGGTAACTTCTTATCCTAGACG-3^′^, reverse primer of *AtVKOR*, 5^′^- CTGAGAGTTTTGTGCTAAGG-3^′^. Each reaction was carried out in three biological replicates.

### Measurements of Chlorophyll Fluorescence under Different Light

Fully expanded leaves from 8-weeks-old plants were detached and incubated in sterilized water under normal growth light (120 μmol m^-2^ s^-1^) and high light stress (600 μmol m^-2^ s^-1^) for 2 h, respectively. Chlorophyll fluorescence was measured using a pulsemodulated fluorometer (FMS-2, Hansatech, UK) as previously described (Yu et al., 2014). One part of the treated leaves were shielded for dark-adaption for more than 15 min, and then, the dark adapted leaves were used for the measurement of maximum quantum yield of PSII (Fv/Fm; Fv, the variable chlorophyll fluorescence yield, defined as Fm-Fo). The other part of treated leaves were directly used for the measurement of the Φ PSII. The parameters were then calculated as previously described (Krause and Weis, 1991): Fv/Fm = (Fm – Fo)/Fm, ΦPSII = (Fm’ – Fs)/Fm’, and NPQ = (Fm–Fm’)/Fm’. In every experiment, at least six leaves were measured, and three independent experiments were conducted.

### Thylakoid Membrane Protein Preparation and Western Blot Detection

According to the previous description, the thylakoid membranes were prepared from the leaves of the *vkor* mutant line, cysteine-mutant *At*VKORs and *At*VKOR_WT_ transgenic plants under normal growth light or 2 h high light treatment (Lu et al., 2013; Yu et al., 2014). The chlorophyll content was determined in 80% (v/v) acetone according to previous operations (Yu et al., 2014). Protein samples corresponding to equal amounts of chlorophyll were separated through 15% sodium dodecyl sulfate–polyacrylamide gel electrophoresis (SDS–PAGE). Then, the bands of proteins were transferred onto immobilon-P membranes (Millipore, USA) and blotted with specific D1-antibody. The immune-decorated signals were detected by sensitive fluorography with enhanced chemiluminescence (Amersham, Japan). More than three independent experiments were conducted. The D1 amount was quantified by assaying the intensity in the western blot using Image J software.

### H_**2**_O_**2**_ and O_**2**_⋅^-^ Determination

The contents of H_2_O_2_ and O_2_⋅^-^ were determined according to the previous method (Jiang and Zhang, 2002; Clore et al., 2008). Leaves of the *vkor* mutant line, cysteine-mutant, VKORs and VKOR_WT_ transgenic plants under normal growth light and after 2 h high light treatment were ground to a fine power in liquid nitrogen and extracted using 50 mM PBS (pH 7.8). The absorbance was determined at 436 and 530 nm, respectively, and the contents of H_2_O_2_ and O_2_⋅^-^ were calculated according to the standard curve of H_2_O_2_ reagent and NaNO_2_ reagent. Each experiment was carried out in three biological replicates.

## Results

### Replacement of the Conservative Cysteines in *At*VKOR Caused the Pleiotropic Growth Defects in *Arabidopsis*

**FIGURE 1:**
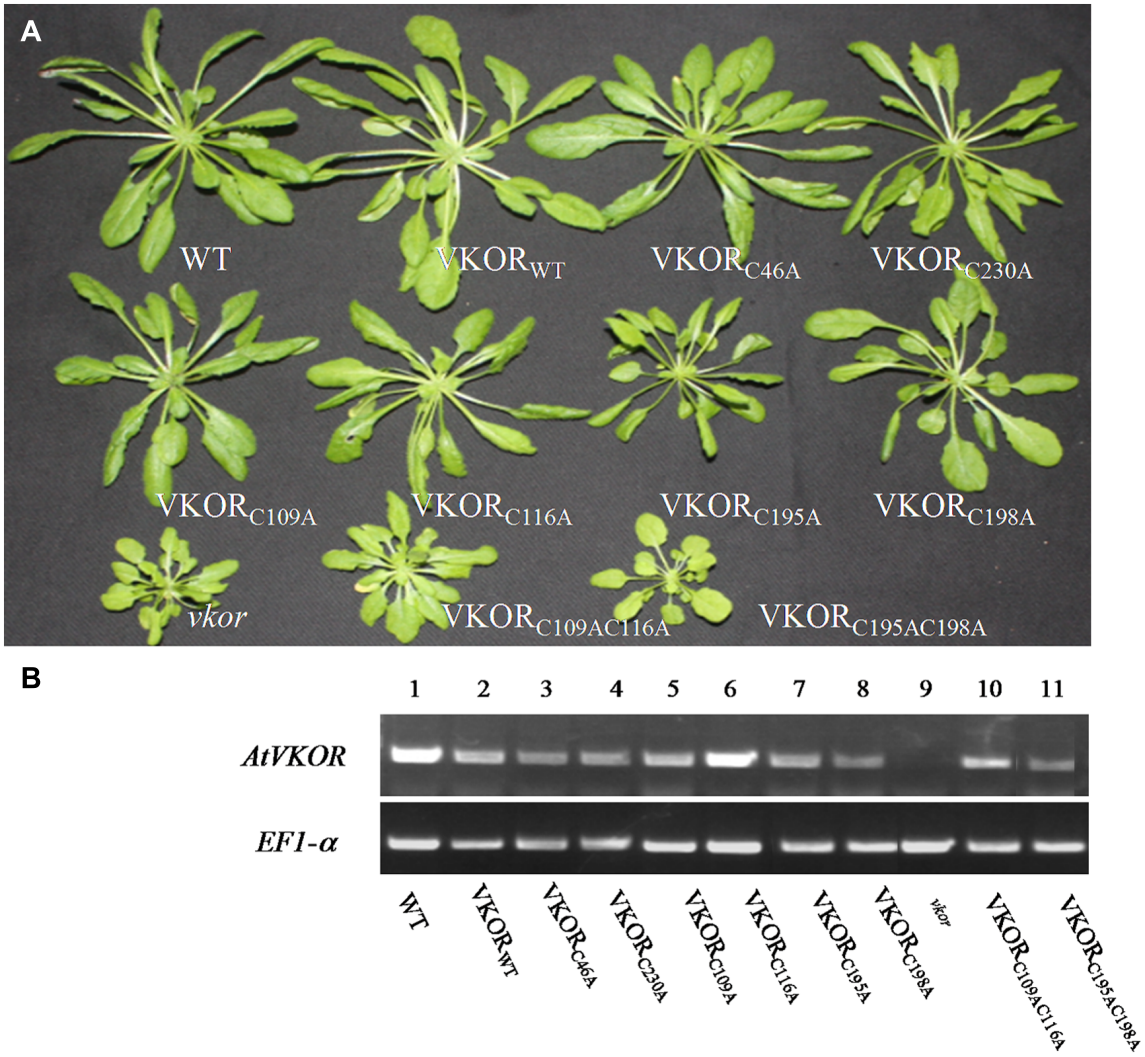
**Characteristics of cysteine-mutant *At*VKORs plants. (A)** The phenotypes of cysteine-mutant *At*VKORs plants; plants were grown on soil for 8 weeks under normal growth conditions. **(B)** Transcriptional level analysis of *AtVKOR* in plants of cysteine-mutant *At*VKORs.

Six cysteines exist in the transmembrane domain of *At*VKOR, including two non-conservative cysteines (Cys46, Cys230) and four conservative cysteines forming two pairs (Cys109/Cys116 and Cys195/Cys198) (Feng et al., 2011). Single/double-cysteine(s) mutant VKORs and wild-type *At*VKOR (VKOR_WT_) were successfully expressed in *vkor* mutant lines, respectively (**Figure 1B**). The representative phenotypes of the *vkor* homozygosities with the insertion of cysteine-mutant VKORs are shown in **Figure 1A**. While the *At*VKOR_WT_ transgenic plants can completely recover the phenotype defects in the *vkor* line, transgenic plants with a single conservative cysteine mutation (*At*VKOR_C109A_, *At*VKOR_C116A_, *At*VKOR_C195A_, *At*VKOR_C198A_) did only partly recover the phenotype defects in the *vkor* mutant lines (**Figure 1A**). When double conservative cysteines were mutated to alanines, the *At*VKOR_C109AC116A_ and *At*VKOR_C195AC198A_ transgenic plants completely lost the ability to compensate for these defects in the *vkor* mutant, displaying significantly stunted growth, smaller sized leaves, and delayed flowering, quite similar to the defects of the *vkor* mutant line. However, the transgenic plants with non-conservative cysteine mutations (*At*VKOR_C46A_, *At*VKOR_C230A_) showed no obvious difference, compared with WT plants and *At*VKOR_WT_ plants.

**FIGURE 2:**
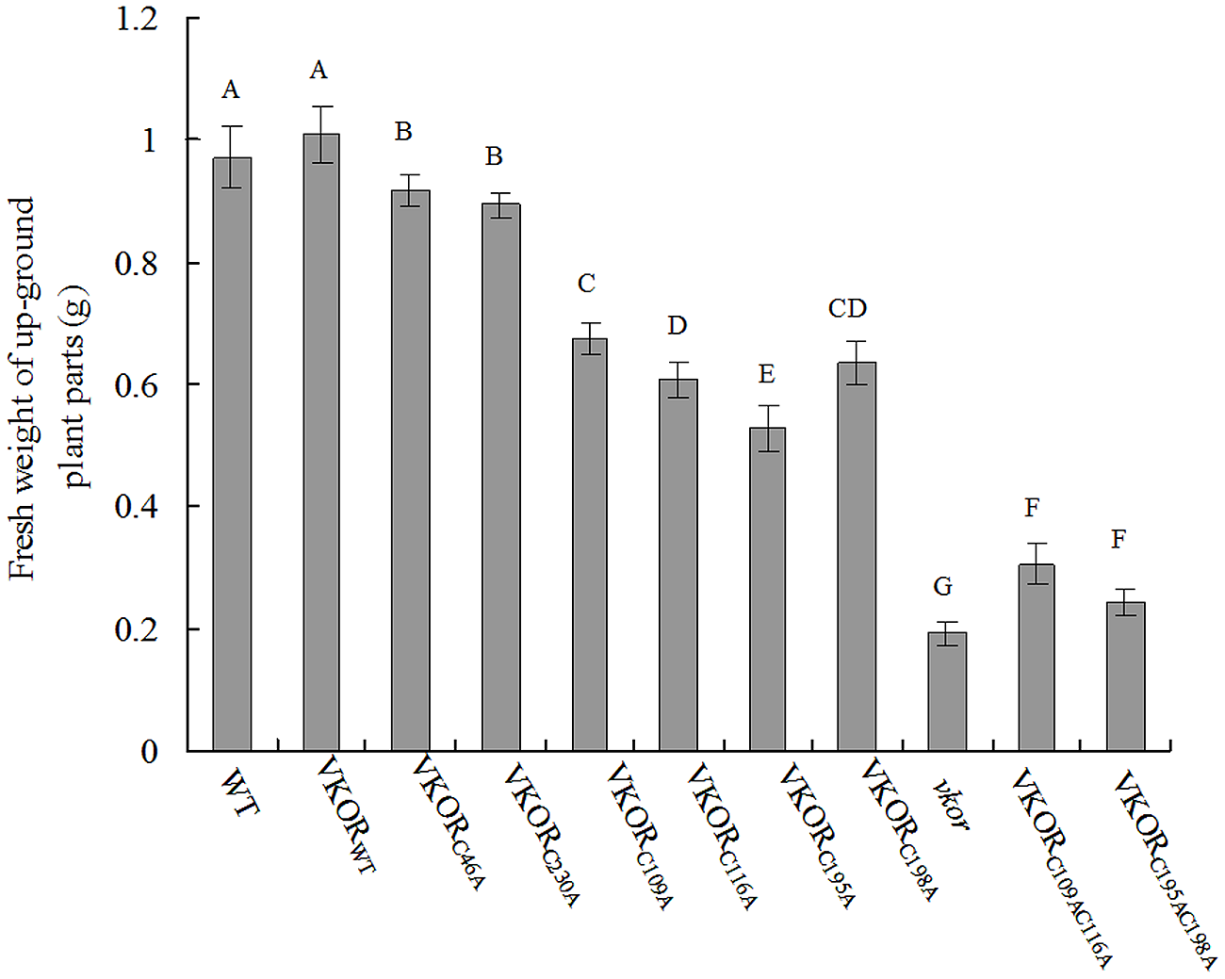
**The biomass of cysteine-mutant *At*VKORs plants.** The fresh weights (FW) of up-ground parts of WT, *vkor* mutant,* At*VKOR_WT,_ and cysteine-mutant VKORs plants were measured. The experiments were repeated at least three times and more than three plants were used each time. The error bars indicated the SD. Significant differences are determined using one-way ANOVA and Ducan’s Multiple Range Test, as indicated with different letters at *P* < 0.05 significance level.

The changes of biomass were further detected in the transgenic plants. The fresh weight (FW) of transgenic plants with double-cysteine mutations (*At*VKOR_C109AC116A_ and *At*VKOR_C195AC198A_) decreased significantly, only about 30% of that of WT and a little more than that of *vkor* deficient lines (**Figure 2**). As to transgenic plants with the non-conservative cysteine mutations (*At*VKOR_C46A_ and *At*VKOR_C230A_), the FW decreased a little, about 95% of that of WTs (**Figure 2**). The changes of biomass were consistent with the phenotypes observed above, further confirming indispensability of the conservative cysteines of *At*VKOR in photosynthetic growth of plants.

The effects of the non-conservative and conservative cysteines of *At*VKOR to *Arabidopsis* phenotypes were consistent with their effects to the formation of a disulfide bond in *E. coli*, although a slight difference exists. The mutations of two non-conservative cysteines do not affect the function of *At*VKOR in catalyzing the formation of a disulfide bond, but each single or double mutation of conservative cysteines absolutely leads to the loss of the catalytic ability by checking the motility and β-galactosidase activity in *E. coli* (Feng et al., 2011). Unlike the phenotype changes of bacteria, in* Arabidopsis*, transgenic plants with a single conservative cysteine VKORs mutation partly recovered the deficient phenotypes, which revealed that a single cysteine mutation did not break down the electron transferring in plants (**Figure 1**). Among the four single mutations, the growth of *At*VKOR_C195A_ was the worst. Combined with the results of double mutations, we supposed the cysteine 195 was directly involved in electron transferring. Characterizations of plant phenotypes demonstrated that all four conservative cysteines could form in pairs when *At*VKOR played functions in photosynthetic growth in *Arabidopsis*.

### PSII Activities Were Inhibited in the Transgenic Plants With Conservative Cysteine VKORs Mutation Both Under Normal Growth Light and High Light

*Arabidopsis* VKOR has been proven to be required for the assembly of PSII; Karamoko et al., 2011), we wondered whether the cysteines in *At*VKOR also affected the activities of PSII. The maximal quantum yield of PSII (Fv/Fm), an indicator for the efficiency of PSII photochemistry (Lu et al., 2011), was determined by a chlorophyll fluorescence measurement in the WT plants, *vkor* mutant line, cysteine-mutant VKORs, and *At*VKOR_WT_ transgenic plants under growth light (120 μmol m^-2^ s^-1^). As shown in **Table 1**, no significant difference in the Fv/Fm ratio was observed among the *At*VKOR_C46A_, *At*VKOR_C230A_, *At*VKOR_WT_ transgenic plants, and WT plants (**Table 1**), indicating that wild-type VKOR as well as the mutation of non-conservative cysteines in the VKOR domain were all sufficient to restore photosynthetic efficiency under normal growth conditions. However, the ratios of Fv/Fm in *At*VKOR_C109AC116A_ and *At*VKOR_C195AC198A_ plants (respectively, 0.662 and 0.673) were dramatically lower than that in the *At*VKOR_WT_ plant (averagely 0.866), indicating the decreased activity of the reaction centers of PSII due to the redox-inactive mutation of conservative cysteines in the VKOR domain. Moreover, a single mutation at Cys195 could also arouse a significant decrease in Fv/Fm (averagely 0.699). A similar trend of actual PSII photochemical efficiency (ΦPSII) was observed in the investigated plants (**Table 1**). These low chlorophyll fluorescence parameters in transgenic plants with mutations of conservative cysteine VKORs were correlated with the impaired photosynthesis and reduced activities of PSII.

**Table 1:** Photosynthetic characterization of wild-type *Arabidopsis, vkor* mutant lines, and transgenic cysteine-mutant VKORs and VKOR_**WT**_ plants under normal growth light vs. high light.

Plants	Normal growth light (120 μmol m^-2^ s^-1^)			2 h High light(600 μmol m^-2^ s^-1^)		
	Fv/Fm	ΦPSII	NPQ	Fv/Fm	ΦPSII	NPQ
WT	0.865 ± 0.005 A	0.769 ± 0.008 A	1.204 ± 0.063 A	0.718 ± 0.008 A	0.436 ± 0.034 A	1.604 ± 0.013 A
VKOR_WT_	0.866 ± 0.004 A	0.766 ± 0.013 A	1.201 ± 0.091 A	0.725 ± 0.005 A	0.445 ± 0.008 A	1.612 ± 0.008 A
VKOR_C46A_	0.861 ± 0.004 A	0.732 ± 0.013 AB	1.151 ± 0.064 A	0.712 ± 0.01 AB	0.421 ± 0.02 AB	1.524 ± 0.036 A
VKOR_C230A_	0.86 ± 0.002 A	0.729 ± 0.02 AB	1.166 ± 0.061 A	0.702 ± 0.014 AB	0.418 ± 0.017 AB	1.516 ± 0.038 A
VKOR_C109A_	0.817 ± 0.013 B	0.709 ± 0.02 B	1.026 ± 0.035 B	0.618 ± 0.026 BC	0.353 ± 0.018 B	1.23 ± 0.102 B
VKOR_C116A_	0.806 ± 0.026 B	0.713 ± 0.046 B	1.012 ± 0.013 B	0.624 ± 0.022 C	0.354 ± 0.022 B	1.225 ± 0.102 B
VKOR_C195A_	0.699 ± 0.051 B	0.653 ± 0.02 B	0.998 ± 0.011 B	0.605 ± 0.027 DE	0.345 ± 0.016 BC	1.219 ± 0.094 B
VKOR_C198A_	0.746 ± 0.047 B	0.696 ± 0.037 B	1.008 ± 0.014 B	0.617 ± 0.024 D	0.35 ± 0.011 BC	1.221 ± 0.095 B
*vkor*	0.565 ± 0.017 D	0.396 ± 0.071 D	0.905 ± 0.012 C	0.441 ± 0.019 F	0.219 ± 0.017 D	1.043 ± 0.068 C
VKOR_C109AC116A_	0.662 ± 0.025 C	0.514 ± 0.019 C	0.945 ± 0.01 BC	0.489 ± 0.026 DE	0.278 ± 0.017 C	1.112 ± 0.093 BC
VKOR_C195AC198A_	0.673 ± 0.025 C	0.525 ± 0.015 C	0.949 ± 0.14 BC	0.493 ± 0.018 E	0.289 ± 0.014 C	1.116 ± 0.089 BC

The number is reported as the mean ± SD of three independent measurements for each plant. Significant differences among wild-type *Arabidopsis* (WT), *vkor* mutant lines, and transgenic cysteine-mutant VKORs and VKOR_*WT*_ plants are determined using one-way ANOVA and Ducan’s Multiple Range Test, indicated with different letters at *P* < 0.05 significance level.

Excess light has negative impacts on plant photosynthesis, and previous research shows that the mutant line of *vkor* is sensitive to high light (Yu et al., 2014). Our results demonstrated that high irradiance (HL, 600 μmol m^-2^ s^-1^ for 2 h) increased the differences on Fv/Fm and ΦPSII among double conservative cysteine-mutant VKORs and *At*VKOR_WT_ plants, compared with normal growth light (GL, 120 μmol m^-2^ s^-1^), as shown in **Table 1**, suggesting that the photoinhibition in *At*VKOR_C109AC116A_ and *At*VKOR_C195AC198A_ plants was further aggravated when exposed to high light.

To avoid photodamage, photosynthetic organisms have developed various photoprotective mechanisms to resist photooxidative damage and to repair damaged protein components (Nishiyama et al., 2001; Murata et al., 2007). One important pathway is to minimize excitation pressure on PSII by thermal dissipation, which can be reflected by the value of NPQ. In this investigation, we found that the NPQ values were lower in the *At*VKOR_C109AC116A_ (averagely 0.945, GL; averagely 1.112, HL) and *At*VKOR_C195AC198A_ (averagely 0.949, GL; averagely 1.116, HL) plants compared with *At*VKOR_WT_ (averagely 1.201, GL; averagely 1.612, HL) plants under different illumination (**Table 1**), suggesting a low capability in the dissipation of excess light. Previous investigations have proved that the *npq1* mutant exhibits greatly reduced NPQ with deficient VDE, a vital enzyme in xanthophyll cycle (Niyogi et al., 1998; Han et al., 2010). *At*VKOR/LTO1 has been proven to be related with the xanthophyll cycle, one important mechanism to dissipate excess thermal energy (Yu et al., 2014). We supposed that mutations of conservative cysteines of *At*VKOR affected the cycle of xanthophyll and resulted in the impaired photoprotection for thermal dissipation.

### Levels of D1 Protein Decreased in the Transgenic Plants with a Conservative Cysteine VKORs Mutation

**FIGURE 3:**
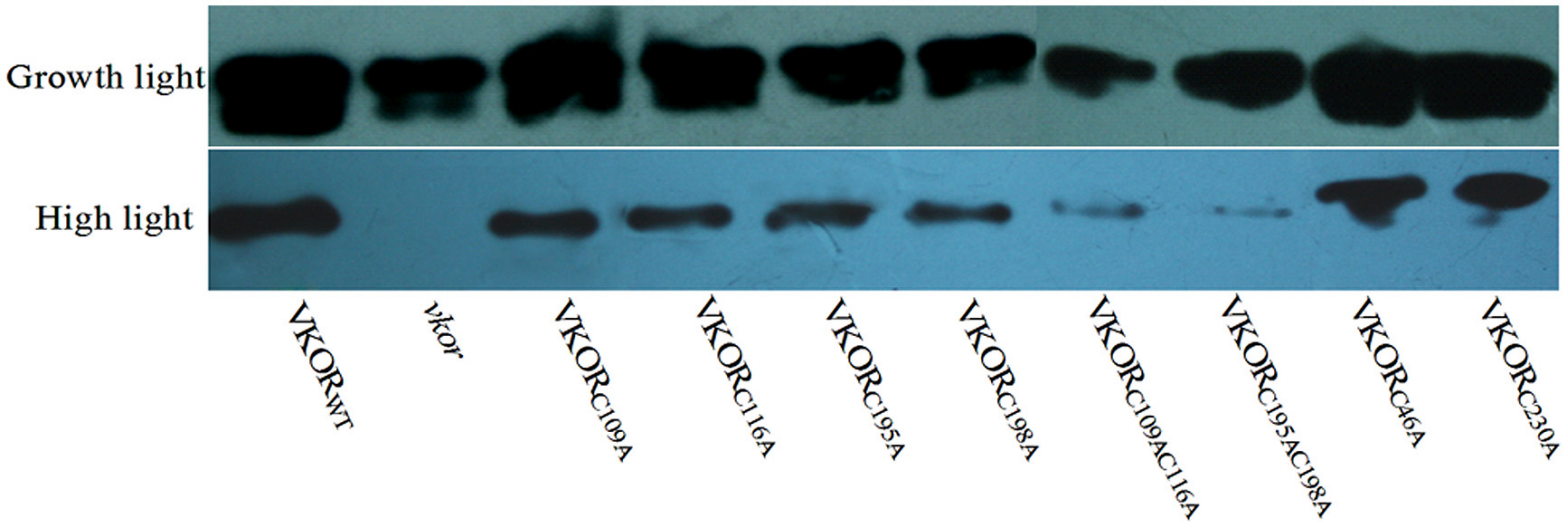
**Immunoblot analysis of D1 accumulation in cysteine-mutant VKORs transgenic plants under growth light or in high light.** Thylakoid membrane proteins were extracted from the leaves of *At*VKOR_WT_, *vkor* mutant and cysteine-mutant VKORs plants. The immunoblot was detected by D1-antibody. Growth light: 120 μmol m^-2^ s^-1^; High light: 600 μmol m^-2^ s^-1^ for 2 h.

**FIGURE 4:**
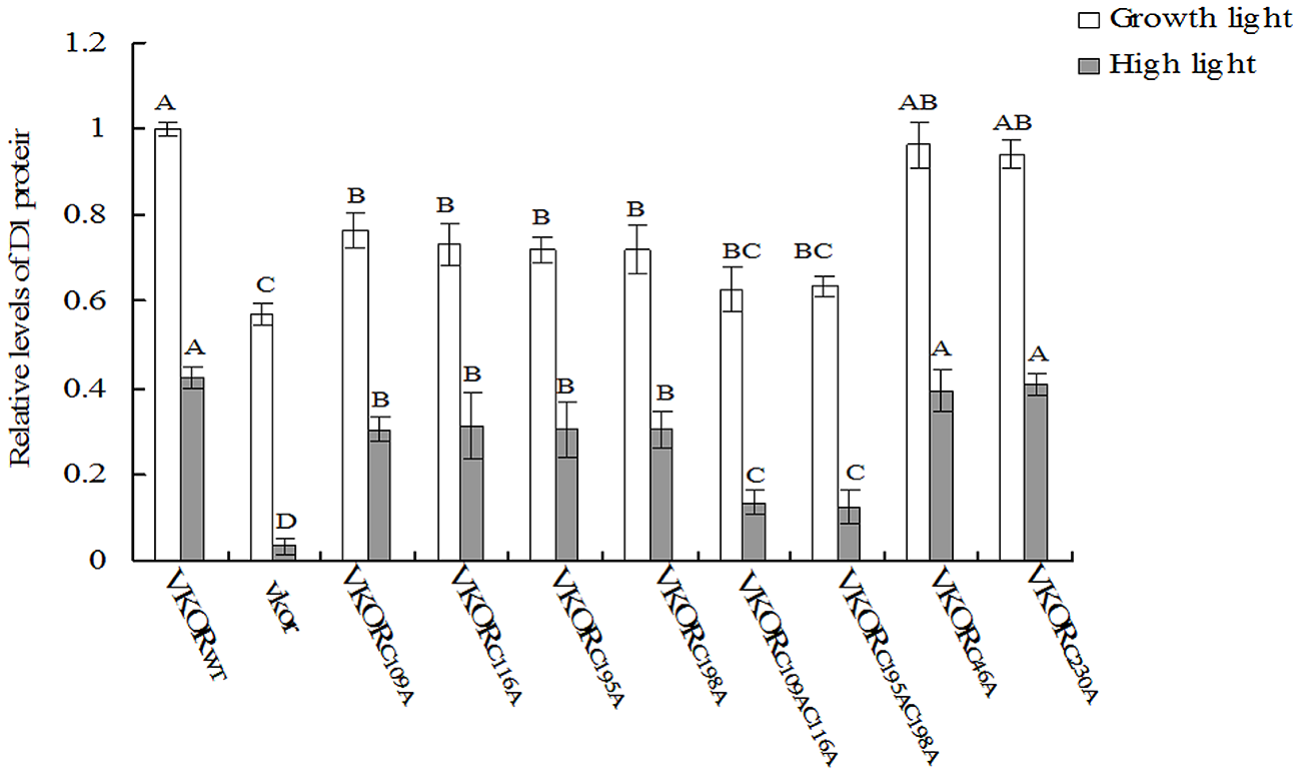
**Quantified analysis of D1 immunoblot.** Signals of immunoblot were quantified using the ImageJ program. The value of D1 accumulation in *At*VKOR_WT_ transgenic plants under growth light was adjusted to one. The relative levels of D1 accumulations in cysteine-mutant VKORs plants compared to VKOR_WT_ plants were, respectively, calculated. The error bars indicated the SD. Significant differences are determined using one-way ANOVA and Ducan’s Multiple Range Test, indicated with different letters at *P* < 0.05 significance level. Growth light: 120 μmol m^-2^ s^-1^; High light: 600 μmol m^-2^ s^-1^ for 2 h.

The turnover of D1 protein is one of photoprotective processes in PSII under high light stress (Nishiyama et al., 2001; Huesgen et al., 2006). Under normal growth light, the levels of D1 protein in transgenic plants with mutant *At*VKORs of conservative cysteine were decreased, especially in double-cysteine mutants *At*VKOR_C109AC116A_ and *At*VKOR_C195AC198A_ (**Figures 3** and **4**). High irradiance increased the decrease extent of D1 protein, and only trace amount of D1 accumulation could be detected in the *vkor* mutant line and double-cysteine mutant plants, which is consistent with the previous result that the deficiency of *At*VKOR accelerates the degradation of D1 protein (Yu et al., 2014). Little difference was observed in the level of D1 protein among the transgenic plants with mutant *At*VKORs of non-conservative cysteine and *At*VKOR_WT_ (**Figures 3** and **4**). The results above suggested that the turnover of D1 protein in the repair of photodamaged were impaired due to the mutations of conservative cysteine in the *At*VKOR domain, which are directly related to its oxidoreductase activity.

### Accumulation of ROS Increased in the Transgenic Plants with Conservative Cysteine VKORs Mutation

**FIGURE 5:**
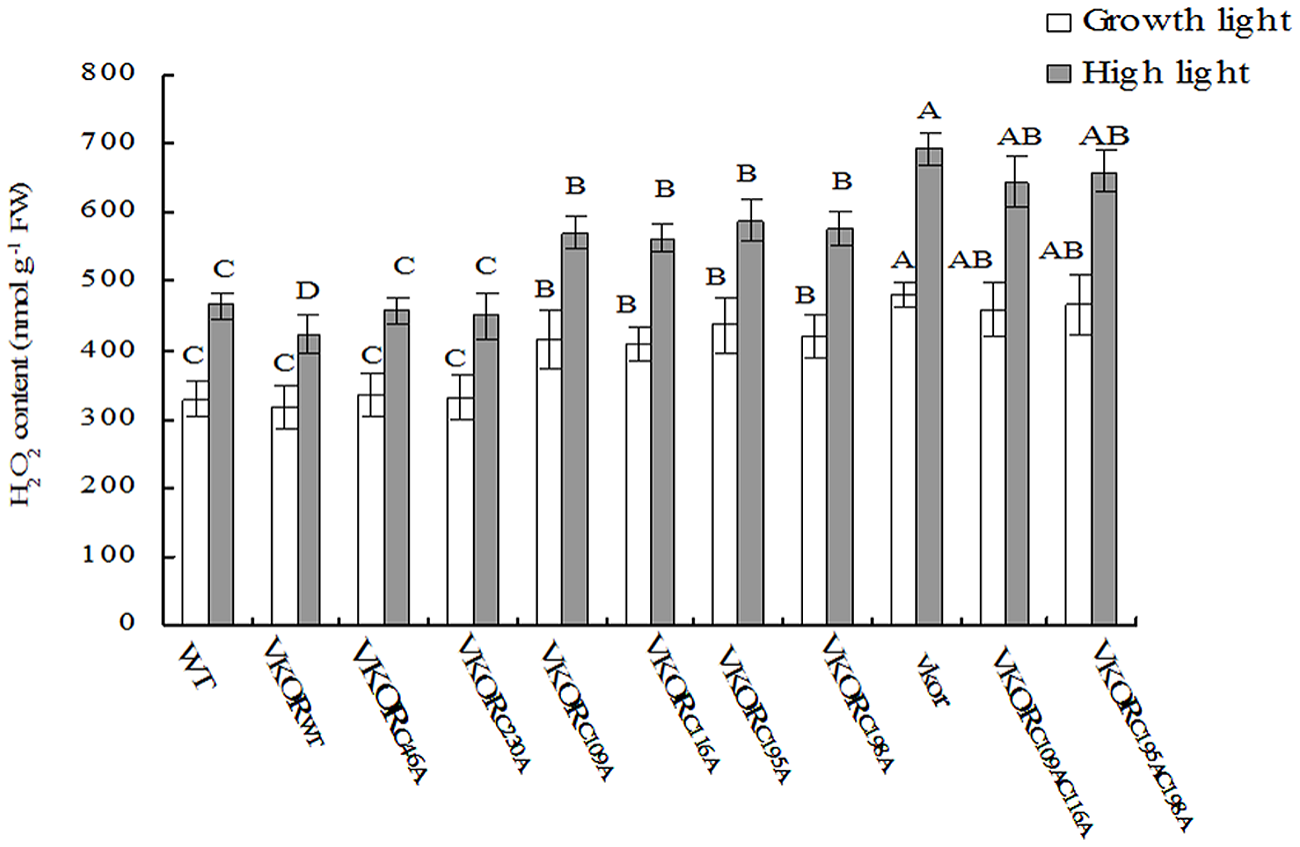
**Levels of H_**2**_O_**2**_ in cysteine-mutant VKORs transgenic plants under growth light or in high light.** Quantitative accumulations of H_2_O_2_ were, respectively, detected in 6-weeks-old leaves from WT, *vkor* mutant,* At*VKOR_WT,_ and cysteine-mutant VKORs plants under growth light or in high light. The error bars indicated the SD. Significant differences are determined using one-way ANOVA and Ducan’s Multiple Range Test, indicated with different letters at *P* < 0.05 significance level. Growth light: 120 μmol m^-2^ s^-1^; High light: 600 μmol m^-2^ s^-1^ for 2 h.

**FIGURE 6:**
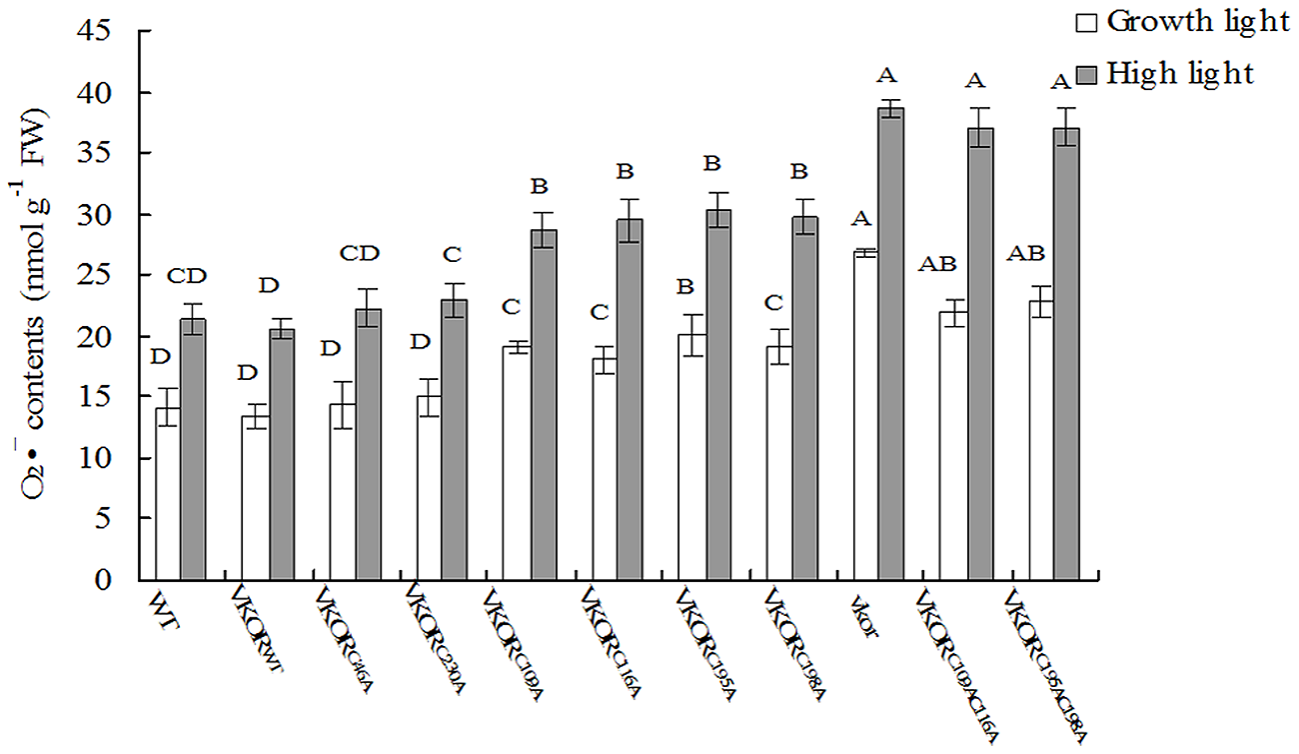
**Levels of O_**2**_⋅^**–**^ in cysteine-mutant VKORs transgenic plants under growth light or in high light.** Quantitative accumulation of O_2_⋅^-^ was, respectively, detected in 6-weeks-old leaves from WT, *vkor* mutant,* At*VKOR_WT,_ and cysteine-mutant VKORs plants under growth light or in high light. The error bars indicated the SD. Significant differences are determined using one-way ANOVA and Ducan’s Multiple Range Test, indicated with different letters at *P* < 0.05 significance level. Growth light: 120 μmol m^-2^ s^-1^; High light: 600 μmol m^-2^ s^-1^ for 2 h.

In chloroplasts, the damage of PSII assembly is usually associated with the harmful production of ROS, such as H_2_O_2_, O_2_⋅^-^, and singlet oxygen (Cejkova et al., 1998; Murata et al., 2007). Previous studies show that much more H_2_O_2_ and O_2_⋅^-^ are accumulated in the *vkor* mutant than in wild-type plants (Lu et al., 2013). By checking the levels of H_2_O_2_ and O_2_⋅^-^ in transgenic plants, we found that the amounts of ROS in plants with mutations of conservative cysteine VKORs were higher than that of *At*VKOR_WT_ plants under growth light (**Figures 5** and **6**). Under high irradiance, the elevated accumulations of H_2_O_2_ and O_2_⋅^-^ in *At*VKOR_C109AC116A_ and *At*VKOR_C195AC198A_ plants were detected, similar to that of the *vkor* mutant line (**Figures 5** and **6**). As to the transgenic plants of *At*VKOR_C46A_, *At*VKOR_C230A_, the levels of H_2_O_2_ and O_2_⋅^-^ were quite closed to that in *At*VKOR_WT_ plants (**Figures 5** and **6**). The results further indicated that the photoprotection mechanism was damaged in the transgenic plants with mutant *At*VKORs of conservative cysteine.

## Discussion

Thylakoid protein *At*VKOR/LTO1, containing a conserved VKOR domain with four conservative cysteines, has been reported to participate in the transmembrane thiol-oxidation as an oxidoreductase *in vitro* and in *E. coli* (Feng et al., 2011; Karamoko et al., 2011). The necessity of the conservative cysteines of the *At*VKOR domain is inferred from the fact that single or double cysteine mutations lead *At*VKOR to losing its function of promoting disulfide bond formation in *E. coli* (Feng et al., 2011). Whether conservative cysteines of* At*VKOR play essential roles in the complicated plant cells needs to be determined.

In this investigation, our results demonstrated that the cysteine-dependent oxidoreductase activity of *At*VKOR was directly related to photosynthetic growth in plant. The *At*VKOR_WT_ transgenic plants can completely recover the phenotype defects in the *vkor* deficient line. Similar to the* At*VKOR_WT_, the mutations of non-conservative cysteines in *At*VKOR domain almost rescued the defects of *vkor* mutant, though there was a decrease of biomass at about 5% of WT. Little effects of non-conservative cysteines on the function of *At*VKOR were further shown by checking photosynthetic parameters, for example Fv/Fm, ΦPSII, and D1 quantity. On the contrary, the double-mutations of each pair of conservative cysteines in *At*VKOR domain did not compensate the defects of *vkor* mutant, displaying a similar phenotype of *vkor* mutant lines. The essentiality of the conservative cysteine residues to activity of VKOR has also been observed in the VKORs from other species (Li et al., 2010; Wang et al., 2011). Mutations of each conservative cysteines of *Mtb*VKOR lead mycobacteria to losing the growing ability in minimal medium (Wang et al., 2011). The structural analysis shows that four conservative cysteines are spatially proximate in the active site of VKOR from* Synechococcus* sp. (Li et al., 2010). Attentively, it has been proven that *At*VKOR has oxidation, reduction, and isomerization activity *in vitro* (Lu et al., 2013). The conservative cysteine of *At*VKOR directly affects electron transferring and is related to the activity of oxidoreductase (Yang et al., 2015). Based on all the investigations, it is quite possible that oxidoreductase activity of *At*VKOR affects the thiol-redox metabolism in chloroplasts and regulates the growth and development of plants.

*At*VKOR is required for the assembly of PSII under growth light, and the deficient *vkor* mutant line is more susceptible to high light stress, compared with WT (Yu et al., 2014). Previous study proves that these defects could be abolished when *AtVKOR*_WT_ is transformed into the* vkor* mutant line, suggesting the phenotype changes are due to the gene (Lu et al., 2013). Our investigation further demonstrated that the stunted growth, the reduced PSII activity, and the aggravated photodamage were related to the damage of *At*VKOR oxidoreductase activity due to the mutation of conservative cysteines in the *At*VKOR domain. Transforming conservative cysteine mutant VKORs to *vkor* mutant line, especially double-cysteine mutant *At*VKORs (*At*VKOR_C109AC116A_ and *At*VKOR_C195AC198A_), could not eliminate the damage, based on the declined fluorescence parameters (Fv/Fm, ΦPSII and NPQ; **Figure 1**; **Table 1**). The high accumulation of ROS in transgenic plants of *At*VKOR_C109AC116A_ and *At*VKOR_C195AC198A_ further verified the severe photodamage.

The damage of PSII is a primary target of photodamage in the photosynthetic apparatus (Nishiyama et al., 2001; Murchie and Niyogi, 2011). To effectively repair the photodamage, diversified photoprotection processes emerge during the evolution of plants (Kirilovsky and Etienne, 1991; Cai et al., 2010; Lunch et al., 2013; Nath et al., 2013). The rapid D1 turnover, a cycle of degradation and re-synthesis of D1 protein, is one of effective photoprotection methods (Ali et al., 2006; Murata et al., 2007). The accumulation of D1 protein depends on the balance of the synthesis and degradation. As to the synthesis of D1 protein, no difference has been found at the transcription level of *PsbA*, the encoding gene of D1, between *vkor* deficient line and WT plants (Lu et al., 2013). However, the effects of *At*VKOR on the translation steps of D1 remain to be elucidated. Recent investigations reveal that the *de novo* synthesis of proteins, particularly D1 protein, can be inhibited by excess ROS at translation level (Nishiyama et al., 2001, 2004, 2006, 2011; Murata et al., 2007; Takahashi et al., 2009). The ROS-induced suppression of protein synthesis is associated with the specific inactivation of elongation factor G via the formation of an intramolecular disulfide bond (Nishiyama et al., 2006, 2011; Murata et al., 2007). Much more ROS was accumulated in plants of mutant *At*VKORs of conservative cysteines in our investigation. So it is quite possible that the synthesis of D1 protein in translation steps would be affected. As to the degradation of D1, when the synthesis of D1 is blocked, the degradation rate of D1 is accelerated in the *vkor* deficient line (Yu et al., 2014). The accelerated degradation of D1 in the *vkor* line may be related to instability of PSII, since *At*VKOR is required for PSII assembly (Karamoko et al., 2011). In this investigation, the low accumulation of D1 in the transgenic plants suggested that the cysteine-dependent activity of *At*VKOR was involved in the D1 turnover-dependent photoprotection mechanism.

One fast response to high irradiance is to dissipate excessive thermal energy by NPQ, the core component of which requires sufficient zeaxanthin produced by the xanthophyll cycle (Lunch et al., 2013). In the xanthophyll cycle, epoxide xanthophyll violaxanthin is rapidly converted via the antheraxanthin to the de-epoxide zeaxanthin, and zeaxanthin can directly participate in the dissipation of excess energy (Murchie and Niyogi, 2011). The key enzyme catalyzing the conversion from antheraxanthin to zeaxanthin is VDE, a thylakoid luminal protein containing essential disulfide bonds (Latowski et al., 2004). Previous investigation has showed that the xanthophyll cycle in the *vkor* mutant line is also impaired under high light, base on the ratio of xanthophyll pigments (Yu et al., 2014). In this investigation, a decline of NPQ both under growth light and high light was observed in transgenic plants of VKOR_C109AC116A_ and VKOR_C195AC198A_, reflecting a low capability to dissipate excessive irradiance. Presumably, this is closely related to the declined activity of VDE, whose disulfide bonds in active site are not correctly formed due to the deficient oxidoreductase activity of *At*VKOR in these transgenic plants. Altogether, our results suggested that conservative cysteines in *At*VKOR domain were related to the oxidoreductase activity of *At*VKOR, and the function was directly involved in photosynthetic growth and PSII activity *in vivo*.

## Author Contributions

Designed the experiments: X-YW, J-JD. Performed the experiments: J-JD, C-YZ, YL, and H-RC. Analyzed the data: J-JD, C-YZ, YL, H-RC, and X-YW. Wrote the paper: J-JD and X-YW.

## Conflict of Interest Statement

The authors declare that the research was conducted in the absence of any commercial or financial relationships that could be construed as a potential conflict of interest.

## Abbreviations

*At*VKOR_CXXA/AXXC/AXXA_ plants: complemented cysteine-mutant VKORs/*vkor* plants
*At*VKOR_WT_ plants: complemented wild-type *At*VKOR/*vkor* plants
H_2_O_2_: hydrogen peroxide
LTO1: Lumen thiol oxidoreductase 1
NPQ: non-photochemical quenching
O_2_⋅^-^: superoxide anion radical
ΦPSII: actual PSII efficiency
PSII: photosystem II
qRT-PCR: quantitative reverse transcription-polymerase chain reaction
ROS: reactive oxygen species
VDE: violaxanthin de-epoxidase
VKOR: vitamin K epoxide reductase
WT: wild-type *Arabidopsis*
